# Awake craniotomy during pregnancy: A systematic review of the published literature

**DOI:** 10.1007/s10143-023-02187-x

**Published:** 2023-11-01

**Authors:** Mohammad Mofatteh, Mohammad Sadegh Mashayekhi, Saman Arfaie, Hongquan Wei, Arshia Kazerouni, Georgios P. Skandalakis, Ahmad Pour-Rashidi, Abed Baiad, Lior Elkaim, Jack Lam, Paolo Palmisciano, Xiumei Su, Xuxing Liao, Sunit Das, Keyoumars Ashkan, Aaron A. Cohen-Gadol

**Affiliations:** 1 School of Medicine, Dentistry and Biomedical Sciences, Queen’s University Belfast, 97 Lisburn Road, Belfast, BT9 7BL UK; 2 Neuro International Collaboration (NIC), London, UK; 3 Division of Neurosurgery, Department of Surgery, The Ottawa Hospital, Ottawa, ON Canada; 4 Faculty of Medicine, University of British Columbia, Vancouver, BC Canada; 5 Neuro International Collaboration (NIC), Ottawa, ON Canada; 6 Department of Neurology and Neurosurgery, McGill University, Montreal, QC Canada; 7 Department of Molecular and Cell Biology, University of California Berkeley, Berkeley, CA USA; 8 Neuro International Collaboration (NIC), Montreal, QC Canada; 9 Department of 120 Emergency Command Center, Foshan Sanshui District People’s Hospital, Foshan, Guangdong Province China; 10 Memorial University of Newfoundland, St. John’s, Newfoundland and Labrador Canada; 11 First Department of Neurosurgery, Evangelismos General Hospital, National and Kapodistrian University of Athens, Athens, Greece; 12 Department of Neurosurgery, Sina Hospital, Tehran University of Medical Sciences (TUMS), Tehran, Iran; 13 Faculty of Medicine, McGill University, Montreal, QC Canada; 14 Montreal Neurological Institute and Hospital, Department of Neurology and Neurosurgery, McGill University, Montreal, QC Canada; 15 University of Cincinnati College of Medicine, Cincinnati, OH USA; 16 Obstetrical Department, The Second Affiliated Hospital of Guangzhou Medical University, Guangzhou, China; 17 Department of Neurosurgery, Foshan Sanshui District People’s Hospital, Foshan, China; 18 Department of Surgery of Cerebrovascular Diseases, Foshan First People’s Hospital, Foshan, China; 19 Division of Neurosurgery, St. Michael’s Hospital, Toronto, ON Canada; 20 Department of Neurosurgery, King’s College Hospital NHS Foundation Trust, London, UK; 21 Department of Basic and Clinical Neuroscience, Institute of Psychiatry, Psychology and Neuroscience, King’s College London, London, UK; 22 King’s Health Partners Academic Health Sciences Centre, London, UK; 23 School of Biomedical Engineering and Imaging Sciences, Faculty of Life Sciences and Medicine, King’s College London, London, UK; 24 The Neurosurgical Atlas, Carmel, IN USA; 25 Department of Neurological Surgery, Indiana University, Indianapolis, IN USA; 26 Neuro International Collaboration, Indianapolis, IN USA

**Keywords:** Anesthesia, Awake craniotomy, Fetus, Tumor, Pregnancy, Brain mapping

## Abstract

Neurosurgical pathologies in pregnancy pose significant complications for the patient and fetus, and physiological stressors during anesthesia and surgery may lead to maternal and fetal complications. Awake craniotomy (AC) can preserve neurological functions while reducing exposure to anesthetic medications. We reviewed the literature investigating AC during pregnancy. PubMed, Scopus, and Web of Science databases were searched from the inception to February 7th, 2023, following the Preferred Reporting Items for Systematic Reviews and Meta-Analyses (PRISMA) guideline. Studies in English investigating AC in pregnant patients were included in the final analysis. Nine studies composed of nine pregnant patients and ten fetuses (one twin-gestating patient) were included. Glioma was the most common pathology reported in six (66.7%) patients. The frontal lobe was the most involved region (4 cases, 44.4%), followed by the frontoparietal region (2 cases, 22.2%). The awake-awake-awake approach was the most common protocol in seven (77.8%) studies. The shortest operation time was two hours, whereas the longest one was eight hours and 29 min. The mean gestational age at diagnosis was 13.6 ± 6.5 (2—22) and 19.6 ± 6.9 (9—30) weeks at craniotomy. Seven (77.8%) studies employed intraoperative fetal heart rate monitoring. None of the AC procedures was converted to general anesthesia. Ten healthy babies were delivered from patients who underwent AC. In experienced hands, AC for resection of cranial lesions of eloquent areas in pregnant patients is safe and feasible and does not alter the pregnancy outcome.

**Supplementary Information:**

The online version contains supplementary material available at 10.1007/s10143-023-02187-x.

## Introduction

Intracranial pathologies during pregnancy pose a significant challenge to both the mother and fetus [1–3]. A predominance of central nervous system tumors, including gliomas, is observed in females older than 20 years of age, which coincides with the reproductive fertility and child-bearing period in life [4]. As such, some lesions such as choriocarcinomas, meningiomas, and pituitary adenomas may be specifically linked to pregnancy [2, 5].

With extensive physiological changes occurring during pregnancy, some may alter the growth of already existing intracranial lesions or unmask signs and symptoms of previously unknown ones [6]. Another life-threatening concern is vasogenic cerebral edema secondary to growing intracranial tumors which may lead to a sudden increase in intracranial pressure [7]. For these reasons, neurosurgical interventions are recommended in pregnant patients with malignant tumors regardless of gestational age [8]. Treatments of brain lesions, including chemotherapy, radiation, and surgical resection under general anesthesia can have potentially teratogenic and fatal consequences for the fetus, thereby complicating the obstetric management of pregnant patients [4, 9].

Neurosurgical interventions for intracranial pathologies are well-tolerated by both the mother and the fetus [10]. Most studies have investigated the neurosurgical treatment of pathologies under general anesthesia in pregnancy [11–15]. Lesions in eloquent areas require awake craniotomy (AC) for real-time monitoring of neurological functions, including motor, language, and vision [16–20]. Recent studies show AC is a safe procedure with fewer post-operative complications such as stress and anxiety for patients [21].

AC results in lower neurological deficits while enhancing the chance of achieving maximal macroscopic resection in different pathologies, including glioblastoma [22–24], glioma [23, 25–28], meningioma [29, 30], and aneurysms [31]. The extent of tumor resection is correlated significantly with the patient’s survival [32]. In addition, AC avoids medication usage for general anesthesia which may otherwise have teratogenic effects [33, 34]. Furthermore, undergoing neurosurgery is a significant physical and mental undertaking. Experiencing psychological distress, directly or indirectly, during pregnancy, can cause adverse birth outcomes, such as preterm birth [35]. Complications associated with brain surgery under general anesthesia complicate the health of a childbearing person and fetus [36, 37]. Therefore, maintaining maternal psychological well-being is of paramount importance.

Multiple factors, such as maternal and fetal well-being, patient’s willingness, and available expertise influence the choice between craniotomy under general anesthesia or while awake. Given the benefits of AC, it would be intuitive to assume that AC would be performed more routinely in pregnancy. However, a unified synthesis of the literature on AC for pregnant patients has yet to be performed. This is the first systematic review that fills the gap in the treatment of brain lesions in pregnant people using AC.

## Materials and methods

### Inclusion and exclusion criteria

Articles were eligible for our systematic review if they met the following criteria: (1) original articles (2) English only, (3) articles involving human subjects only, (4) AC operation during any trimester of pregnancy, and (5) sufficient data could be extracted from an available article in databases. The exclusion criteria were defined as (1) studies with mixed reports on pregnant and non-pregnant patients, (2) sufficient data could not be extracted, and (3) AC and other techniques used without individual patient data differentiation.

### Information sources and search strategy

This systematic review was conducted based on the Preferred Reporting Items for Systematic Reviews and Meta-Analysis (PRISMA) guidelines [38] to identify published literature on AC during pregnancy, and a separate protocol was not established. We conducted our electronic searches using the PubMed, Scopus, and Web of Sciences databases from inception to February 7th, 2023 for relevant articles. The following Boolean terms were used for the search: (“awake craniotomy” OR “awake brain surgery” OR “awake neurosurgery” OR “awake brain mapping” OR “awake tumor resection” OR “craniotomy while awake”). Details of search terms for each database are shown in Supplementary Table 1.

### Selection process

At least two authors (M.M., M.S.M., and S.A.) independently screened the titles and abstracts of initial search results. Following the title and abstract screening, relevant papers were selected for full-text screening. In addition, M.M., M.S.M., and S.A. screened for relevant articles via the reference lists of selected articles to ensure additional appropriate articles were not excluded.

### Data extraction

At least two authors (M.M., M.S.M., S.A. and A.K.) interchangeably extracted data from each selected paper. All four authors (M.M., M.S.M., S.A. and A.K.) further validated the extracted data independently of the author who initially completed the extraction. The following data of interest were extracted from the final articles: first author, publication year, title, journal, country, study objectives, study period, study design, single/multi-centre, condition treated, anesthesia protocol, primary sedation, and analgesia, mean AC operation time, follow-up period, total patient number, whether AC and GA were compared directly, number of patients, number of fetuses, mean age of patients, pathology, presentation, surgical position, gestational age at diagnosis, gestational age at craniotomy, pregnancy term, delivery method, hospitalization length, AC intraoperative complications, neonatal outcome, lesion hemisphere, lesion location, eloquent area lesion and mapping, intraoperative fetal monitoring, pre-operative localization, AC extent of resection, conversion to GA, pre-operative neurological symptoms, post-operative complications, post-operative neurological deficits resolved, and main outcome reported. Study risk of bias assessment was not used given that no meta-analyses were conducted. All calculations were done on Microsoft Excel (version 2016; Microsoft, Redmond, WA, USA). Independently abstracted data were managed on Microsoft Excel Spreadsheet (version 2016; Microsoft, Redmond, WA, USA).

## Results

### Study selection

**Fig. 1:**
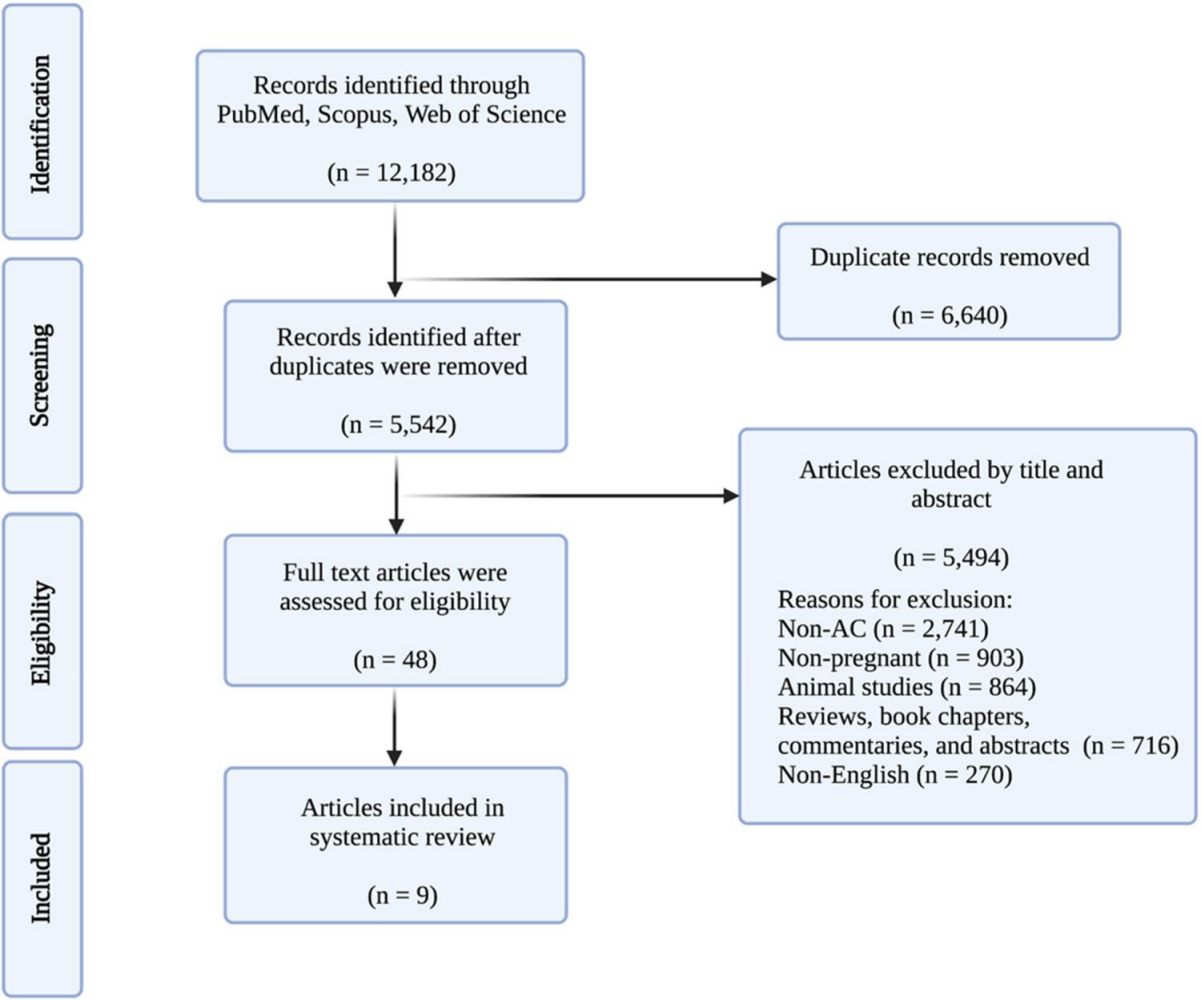
Preferred Reporting Items for Systematic Reviews and Meta-Analyses (PRISMA) flowchart demonstrating the search, screen, inclusion, and exclusion process for the current study. AC, awake craniotomy

Our search yielded 12,182 results across three databases: PubMed (*n* = 3,832), Scopus (*n* = 4,750), and Web of Science (*n* = 3,600). Duplicates (*n* = 6,640) were removed after the search was completed. The remaining studies (*n* = 5,542) were screened based on their titles and abstracts, and studies not relevant to our systematic review were removed (*n* = 5,494). Major reasons for exclusion based on the title and abstract screening were classified as studies irrelevant to AC (*n* = 2,741), studies without pregnant patients (*n* = 903), animal studies (*n* = 864), literature reviews, book chapters, abstracts, and commentaries (*n* = 571), non-English (*n* = 270), commentaries and letters to editors (*n* = 145). If more than one reason was applicable for exclusion, only one criterion was recorded. The remaining articles (*n* = 48) were then fully read for eligibility criteria. Nine studies met the eligibility criteria based on the inclusion and exclusion criteria above and were included in the final review (Fig. 1).

The articles’ information is summarized in Supplementary Table 2. Of the nine studies included, five (55.6%) were from the USA [39–43], followed by Japan [44], Oman [45], Poland [46], and Pakistan [47], with one (11.1%) study each.

### Study characteristics

All studies reported one case. Overall, nine patients with ten fetuses (one twin pregnancy reported by Meng et al. [41]) were included (Table 1). Apart from one study (11.1%) which used AC for pseudoaneurysm [42], all other papers (88.9%) treated tumors via AC [39–41, 43–47]. The awake-awake-awake approach was the most common protocol used in seven studies (77.8%) [40–43, 45–47]. One study (11.1%) used the asleep-awake-asleep protocol [44], and another (11.1%) did not specify their protocol [39].

**Table 1:** An Overview of Studies’ Characteristics and Procedure Details

Study	Study design	Single/multi center	Condition treated	Patients number	Fetus number	AC protocol	Primary sedation and analgesic
Abd-Elsayed et al. 2013 [39]	Retrospective	Single center	Tumor	1	1	NS	Anesthesia induction and maintenance: propofol/alfentanil (dose NS)
Handlogten et al. 2015 [40]	Case study	Single center	Tumor	1	1	Awake-awake-awake	Local anesthesia: 0.25% bupivacaine with 1:200,000 epinephrine Sedation: initial infusion of 0.4 to 0.5 μg/kg/h of dexmedetomidine without a loading dose Intermittent boluses of propofol at 10–30 mg or fentanyl 25 μg administered as needed for stimulating events, such as urinary catheter placement
Meng et al. 2016 [41]	Case study	Single center	Tumor	1	2	Awake-awake-awake	Local anesthesia: 1:1 mixture of 0.5% lidocaine and 0.25% bupivacaine Conscious sedation: propofol (25—60 mcg/kg/min) and remifentanil (0.04—0.14 mcg/kg/min)
Hedayat et al. 2017 [42]	Case study	Single center	Pseudoaneurysm	1	1	Awake-awake-awake	Fentanyl and Diprivan (dose NS)
Kamata et al. 2017 [44]	Case study	Single center	Tumor	1	1	Asleep-awake-asleep	Local anesthesia: 40 ml of 0.3% ropivacaine and 10 ml of 1% lidocaine with 0.01% epinephrine Induction: fentanyl 125 μg and thiopental 375 mg Maintenance: sevoflurane and remifentanil at 0.3 μg/kg/min Conscious sedation: dexmedetomidine started at 1.0 ug/kg/hr and continued at 0.7 μg/kg/hr after 20 min Surgical site closure: fentanyl 225 μg and droperidol 100 μg
Al Mashani, 2018 [45]	Case study	Single center	Tumor	1	1	Awake-awake-awake	Local anesthesia: 0.25% bupivacaine and 1% lidocaine (1:1 mixture) Conscious sedation: low-dose propofol (50 mcg/kg/min), remifentanil (0.1 mcg/kg/min), and dexmedetomidine (0.5 mcg/kg/min). Propofol was stopped and dexmedetomidine was reduced before neurophysiologic testing (0.3 mcg/kg/min)
Pawlik et al. 2018 [46]	Case study	Single center	Tumor	1	1	Awake-awake-awake	Local anesthesia: 15 ml of 1% ropivacaine and 15 ml of 1.0% lidocaine with 1:40,000 epinephrine Conscious sedation: dexmedetomidine at 0.4 to 0.7 mcg/kg/hr without a loading dose, and remifentanil of 1–2 ng/ml. Dexmedetomidine infusion was increased to 1.0 mcg/kg/hr and remifentanil to 3–4 ng/ml during mitigating events (such as scalp block infiltration, and head immobilization)
Kumar et al. 2020 [47]	Case study	Single center	Tumor	1	1	Awake-awake-awake	NS
Gunasekaran et al. 2022 [43]	Case study	Single center	Tumor	1	1	Awake-awake-awake	Local anesthesia: 2% lidocaine with 1:10,000 epinephrine and 0.5% ropivacaine mixed 1:1 Conscious sedation: remifentanil, dexmedetomidine, and propofol

*NS* not specified

### Medications

As the use of propofol in pregnant patients is prohibited, Kamata et al. [44] used sevoflurane and remifentanil for general anesthesia until the first scan of iMRI, and they subsequently re-induced dexmedetomidine when tumor removal had been accomplished. Also, they used prophylactic antiemetics and antacids to prevent intraoperative vomiting. Similarly, Hedayat and colleagues [42] used dexamethasone and ondansetron to provide antiemetic effects. Furthermore, they used ranitidine and oral sodium citrate to decrease the acidity of gastric contents to avoid aspiration.

The mean age of patients was 29.8 ± 4.5 years old, with the youngest patient being 24 [43], and the oldest, 40 [39] (Table 2). One study (11.1%) did not specify the patient's age [42]. Astrocytoma was the most common pathology reported in three (33.3%) patients. Glioma was the most represented pathology in six (66.7%) patients [39–41, 43, 44, 46]. Pseudoaneurysm [42], and meningioma [47] were reported in one study (11.1%) each. One study (11.1%) did not specify the pathology [45]. The shortest operation time was two hours [43], whereas the longest procedure took 8 h and 29 min to complete [40]. However, it is plausible that different studies used various time intervals as operation time; for example, Pawlik et al. [46] stated that the total time spent in the operation theatre was 4 h and 40 min. Four studies (44.4%) did not report their operation time [39, 41, 42, 45]. Lateral/semi-lateral was the common surgical position in four (44.4%) studies [41, 43, 45, 46]. The supine position was employed in two (22.2%) studies [40, 44], and three (33.3%) studies did not specify their surgical position [39, 42, 47].

**Table 2:** Patient Characteristics Summary

Study	Age (year)	Weight and height	Pathology	Presentation	Past medical history	Operation time	Surgical position	Follow-up
Abd-Elsayed et al. 2013 [39]	40	NS	Glioma	Seizure	NS	NS	NS	NS
Handlogten et al. 2015 [40]	27	70 kg, 172 cm	Anaplastic oligoastrocytoma (WHO grade III)	New onset seizure, mild receptive and expressive aphasia	NS	8 h and 29 min	Supine with left uterine displacement	30 months
Meng et al. 2016	31	NS	Anaplastic astrocytoma (WHO grade III)	Word finding difficulty, dysfluency, right upper extremity plegia, and right lower extremity paresis	Two general anesthesia tumor debulking during the same pregnancy at 16 weeks and 28 weeks gestation	NS	Right semilateral	NS
Hedayat et al. 2017 [42]	NS	NS	Cortical pseudoaneurysm of the distal right middle cerebral artery under previous craniotomy site	Intermittent headaches, dizziness, and tingling of hands	Previous craniotomy due to superficially penetrating gunshot wound with complete neurological recovery	NS	NS	NS
Kamata et al. 2017 [44]	30	61.4 kg, 162 cm	Anaplastic astrocytoma (WHO grade III)	Generalized convulsive seizures that were poorly controlled with anti-convulsive therapy	NS	241 min (surgery), 291 min (anesthesia)	Supine with a wedge placed under the right buttock to prevent aortocaval compression	17 months
Al Mashani, 2018 [45]	26	NS	NS	Recurrent seizures for one week	NS	NS	Left lateral position	NS
Pawlik et al. 2018 [46]	31	NS	Astrocytoma (Grade II/III)	Word finding difficulty	NS	4 h and 40 min*	Semilateral	NS
Kumar et al. 2020 [47]	29	53 kg, 153 cm	Meningioma	Left eye blurred vision	Non-significant	4 h	NS	1 week
Gunasekaran et al. 2022 [43]	24	NS	Giant cell glioblastoma (WHO grade IV)	Generalized tonic–clonic seizure, frequent headaches, right leg numbness and weakness, and difficulty with memory and cognition	Systemic lupus erythematosus (1, 100%)	2 h	Left lateral decubitus	1 year

*MRI* magnetic resonance imaging, *NS* not specified, *WHO* World Health Organization.

*Total time spent in the operating theatre.

Six (66.7%) lesions were in the left hemisphere [39–41, 43, 44, 46], whereas the other three (33.3%) were in the right hemisphere [42, 45, 47] (Table 3). The frontal lobe was the most common pathology region reported in four studies (44.4%) [39, 43, 44, 47], followed by frontoparietal regions in two (22.2%) [41, 45]. Temporal [40] and parietal [46] lobes pathologies were each reported in one study (11.1%). One (11.1%) study did not specify the pathologic lobe [42]. Eight studies (88.9%) have reported proximity to eloquent areas such as motor and Wernicke areas as an indication to choose AC instead of GA [40–47]. In addition, 4 studies (44.4%) have specified the potential harm of general anesthesia to the patient and fetus, such as reported acid–base status deterioration caused by prolonged propofol usage in GA for pregnant neurosurgical patients [41, 44, 45, 47, 48]. One study (11.1%) did not specify the indication for choosing AC instead of GA [39]. All studies employed intraoperative fetal monitoring, and seven studies (77.8%) specified fetal heart rate monitoring [39–45]. Two (22.2%) studies did not specify their type of monitoring [46, 47].

**Table 3:** A Summary of Lesion Characteristics and Operation Details

Study	Lesion hemisphere	Lesion location	AC indication	Eloquent area lesion and mapping (*n*, %)	Intraoperative fetal monitoring	Pre-operative localization	AC extent of resection (*n*, %)
Abd-Elsayed et al. 2013	Left (1, 100%)	Frontal lobe (1, 100%)	NS	NS	Yes- Heart rate	NS (2.4 × 2.2 cm)	NS
Handlogten et al. 2015 [40]	Left (1, 100%)	Temporal lobe (1, 100%)	Eloquent language area (Wernicke’s area) lesion	Motor and speech (1, 100%)	Yes- Heart rate and movement	MRI (4.5 × 3.2 × 3.5 cm intracranial mass)	Total/near total (1, 100%)
Meng et al. 2016 [41]	Left (1, 100%)	Frontoparietal white matter (1, 100%)	Avoiding fetus and patient exposure to harmful anesthestic medication, and frontoparietal lesion	Language and sensorimotor (1, 100%)	Yes- Heart rate	MRI (7 × 6 × 5 cm anterior– posterior transverse cranio–caudal)	Subtotal (1, 100%)
Hedayat et al. 2017 [42]	Right (1, 100%)	NS	Cortical lesion	NS	Yes- Heart rate	CT and cerebral angiography	NS
Kamata et al. 2017 [44]	Left (1, 100%)	Frontal lobe (1, 100%)	Avoiding fetus and patient exposure to harmful anesthestic medication, and facilitating full-term birth	Motor (1, 100%)	Yes- Heart rate	MRI (6 cm)	Total/near total (1, 100%)
Al Mashani, 2018 [45]	Right (1, 100%)	Frontoparietal region (1, 100%)	Avoiding fetus and patient exposure to harmful anesthestic medication, and eloquent area (frontoparietal close to motor area) lesion	Motor and speech (1, 100%)	Yes- Tococardiography	MRI (8.2 × 4.5 × 4.6 cm)	NS
Pawlik et al. 2018 [46]	Left (1, 100%)	Parietal lobe (1, 100%)	Eloquent area lesion	Speech (1, 100%)	Yes- NS	MRI (NS)	NS
Kumar et al. 2020 [47]	Right (1, 100%)	Frontal lobe (1, 100%)	Avoiding fetus exposure to harmful anesthestic medication and enhancing recovery	Motor (1, 100%)	Yes- NS	MRI (extra-axial- NS)	Total (1, 100%)
Gunasekaran et al. 2022 [43]	Left (1, 100%)	Frontal lobe (1, 100%)	Eloquent motor area	Motor (1, 100%)	Yes- Heart rate using Doppler	MRI (2.0 × 1.5 × 1.5 cm intracranial mass at the level of the coronal suture)	Total (1, 100%)

*AC* awake craniotomy, *CT* computerized tomography, *MRI* magnetic resonance imaging, *NS* not specified.

### Pre-, intra-, and post-operative monitoring of the fetus

On the day of the AC, Gunasekaran et al. [43] used Doppler to assess fetal cardiac activity, which was indicated as normal. Post-operative Doppler examination also redemonstrated normal fetal activity once the patient was transported to the recovery room. In another study by Kumar and colleagues [47], the fetal heart sounds were monitored pre-operatively by an obstetrician, who administered cyclogest 800 mg rectally as a tocolytic agent.

The mean gestational age at diagnosis was 13.6 ± 6.5 weeks (Table 4). The earliest diagnosis was at two weeks [42], and the latest was at 22 weeks [46] pregnancy. One study (11.1%) did not specify the gestational age at diagnosis [41]. The mean gestational age at craniotomy was 19.6 ± 6.9 weeks with the earliest AC reported on week nine [43], and the latest one at the 30th week [41]. In total, 10 healthy babies were delivered from patients who underwent AC.

**Table 4:** Pregnancy Details and Outcome

Study	Gestational age at diagnosis (week)	Gestational age at craniotomy (week)	Pregnancy term	Delivery method	Hospitalization length	Intraoperative complications	Neonatal outcome
Abd-Elsayed et al. 2013 [39]	18	22	No	NS	NS	None	Viable infant with normal Apgar score
Handlogten et al. 2015 [40]	16	20	Yes	Vaginal	NS	None	Healthy baby delivered vaginally at term
Meng et al. 2016 [41]	NS	30	No- 31 weeks	Under spinal anesthesia on post-operative day 4	NS	NS	Two babies were uneventfully delivered
Hedayat et al. 2017 [42]	2	23	Yes	NS	NS	Unsuccessful primary reconstruction of vessel and aneurysmorrhaphy. Unsuccessful end-to-end bypass	The fetus was delivered at term with no obstetric or neurological complications. Pre, intra, and post-operative monitoring of the fetus's heart rate showed no abnormalities or variabilities
Kamata et al. 2017 [44]	21	27	No- 35 weeks and 2 days	NS	NS	No complications	Healthy baby of 2,137 g delivered. The baby did not show any evidence of medical or developmental concern at 17 months follow-up
Al Mashani, 2018 [45]	10	10	NS	NS	NS	None	NS regarding delivery, did mention that post-operative the fetal status was satisfactory
Pawlik et al. 2018 [46]	22	22	NS	NS	NS	None	No changes in fetal status were observed post-operatively. No fetus or intrauterine volume abnormalities were discovered in ultrasound a day following AC
Kumar et al. 2020 [47]	13	13	NS	NS	3 days	NS	No changes in fetal status post-operatively. Viable heart sounds were heard post-operatively indicating the fetus's viability
Gunasekaran et al. 2022 [43]	7	9	No- the baby was delivered at 34 weeks gestation	Cesarean section	1 day	None	No change in fetal status was observed post-operatively. Doppler monitoring was completed and demonstrated a normal fetal heart rate. The baby was born via cesarian section at 34 weeks gestation due to preterm premature rupture of membranes and concerning fetal monitoring findings (NS). The baby was 5 months old and healthy when the article was written

*AC* awake craniotomy, *NS* not specified.

None of the AC procedures was converted to general anesthesia. A summary of outcomes from each study is summarized in Table 5. Post-operative treatment was specified in three (33.3%) studies [41, 43, 44]. Meng et al. [41] used a partial course of external beam radiotherapy and chemotherapy for their patient. Kamata et al. [44] specified that as pathological examination revealed an anaplastic astrocytoma (WHO grade III), radiotherapy and chemotherapy began two months after delivery. Fractionated radiotherapy was used over 40 days by Gunasekaran et al. [43], and the patient opted out of adjuvant temozolomide treatment. Six (66.7%) studies did not specify the post-operative treatment [39, 40, 42, 45–47].

**Table 5:** Neurological Details and Main Outcome Summary

Study	Conversion to GA	Pre-operative neurological symptoms	Post-operative complications	Post-operative neurological deficits resolved	Post-operative treatment	Main outcome
Abd-Elsayed et al. 2013 [39]	No	NS	Patient deceased 16 months after craniotomy	NS	NS	AC caused no intra or post-operative complications. The baby was delivered with normal Apgar scores. The patient deceased 16 months after the craniotomy
Handlogten et al. 2015 [40]	No	Seizure, expressive & receptive aphafasia	No new neurological deficits	NS	NS	Use of dexmedetomidine and mannitol during AC in a pregnant patient did not lead to notable maternal and fetal adverse effects. There were no intra operative or post-operative complications or newly developed post-operative neurological deficits. The use of mannitol did lead to a transient 30% reduction in internal uterine volume but had no overt maternal or fetal/neonatal adverse effects. A healthy baby was delivered uneventfully at term
Meng et al. 2016 [41]	No	Dysfluency, word-finding difficulty, right upper extremity paralysis, right lower extremities weakness	No immediate post-operative deficits. Receiving hospice care 12 months post-AC. Walking impairment, comprehensible but slurred speech	Significant improvement in fluency with naming and speech comprehension post-operative day 1, but with impaired repetition. The strength of the right arm and leg returned to baseline except for a weak hand grip	Partial course of external beam radiotherapy and chemotherapy	The patient showed significant post-operative day 1 improvement in naming and comprehension, and the strength of the right arm and leg returned to baseline apart from a weak hand grip. No intra operative complications were seen. Two babies (twin) were delivered uneventfully on post-operative day 4 under spinal anesthesia. 12 months post-operative patient is receiving hospice care, with progressive worsening of right leg weakness and slurred speech
Hedayat et al. 2017 [42]	No	Intermittent headaches, dizziness, and hand tingling	No post-operative neurological deficits	NS	NS	AC for management of a pseudoaneurysm in a pregnant female at 23-week gestation was successfully done without any post-operative neurological complications. Pre- and post-operative fetal heart rate monitoring showed no variabilities or abnormalities. The baby was successfully delivered at term. No neurological complications were observed in follow-up visits
Kamata et al. 2017 [44]	No	Generalized convulsive seizures	None	NS	Radiotherapy and chemotherapy 2 months post-delivery	AC for removal of high-grade glioma in a pregnant patient at 27-week gestation was successfully conducted with full tumor resection. No intra operative or post-operative complications were seen. The patient delivered a healthy baby at 35 weeks gestation. At the 17-month follow-up, there was no evidence of tumor recurrence in the patient and no developmental or medical concerns for the child
Al Mashani, 2018 [45]	No	Seizures- unspecified type	None	NS	NS	AC for tumor removal in a pregnant patient at 10-week gestation was completed with no intra operative or post-operative complications. The fetal status was satisfactory intra operatively and post-operatively, there was no information provided regarding the pregnancy
Pawlik et al. 2018 [46]	No	Word finding difficulty	NS	NS	NS	AC for tumor removal in a pregnant patient at 22-week gestation was successfully completed with no intra operative complications. Obstetric abdominal ultrasound was done before, immediately after, and the next day after surgery and all showed no fetal or intrauterine volume abnormalities. AC has neuroprotective effects for the patient and is feasible during pregnancy
Kumar et al. 2020 [47]	No	Left eye blurred vision	NS	Complete resolution of symptoms post-operatively	NS	AC for tumor removal in a pregnant patient at 13-week gestation was successfully completed with complete resolution of symptoms at one-week follow-up. Extensive pre-op psychological preparation was done as part of anesthetic management. Post-operative obstetric review showed fetus viability as evident by fetal heart rate sounds. AC facilitates intraoperative neurological monitoring and provides hemodynamic stability with improved fetal-maternal outcomes
Gunasekaran et al. 2022 [43]	No	Generalized convulsive seizures, subjective right leg numbness and weakness, and difficulty with concentration and memory	NS	NS	Fractionated radiotherapy alone over 40 days. The patient opted out of adjuvant temozolomide treatment	AC for glioblastoma resection in a 24-year-old patient who was pregnant in her first trimester was successfully completed with gross total resection of the tumor. There were no intra operative complications, and after interdisciplinary care with adjuvant radiotherapy, one-year follow-up showed no further disease. There were no post-operative fetal complications, and a healthy baby was born at 34 weeks gestation. At the time the article was written, the baby was 5 months old and in good health. AC is a safe and effective process for resectioning glioblastoma in pregnancy

*AC* awake craniotomy, *NS* not specified.

## Discussion

Significant physiological factors, including hormonal changes, increased blood volume (leading to increased intracranial pressures), and fluid retention may result in increased brain tumor growth and exacerbation of neurological symptoms in pregnant patients [36]. Gliomas and meningiomas are the most common types of adult primary brain tumors among general neurosurgical patients [49] and their growth may be accelerated during pregnancy due to the presence of estrogen and progesterone receptors on tumor cells [50]. In addition, pregnancy can have adverse effects and exacerbate the neurological consequences of tumors such as seizure and brainstem herniation [4]. Tumor growth and exacerbated neurological symptoms can occur in about 30% of pregnant patients with gliomas in the later stages of the pregnancy, probably due to multiple physiological changes, including an increase in blood pressure and endocrine changes causing tumor growth acceleration and edema [12], highlighting the importance of effective measures to tackle such complications. Furthermore, a decrease in the seizure threshold during pregnancy can enhance tumor-associated seizures [12].

In this systematic review, we demonstrated that AC is a feasible and safe procedure for lesions in eloquent areas. Primary malignant brain tumor in pregnant patients can be 2.6–15 per 100,000 [51, 52]. Due to such low incidence, there are insufficient guidelines for the management of intracranial lesions during pregnancy [3, 6, 53, 54], however; the significance of brain tumor in pregnant patients should not be underestimated. Various studies have discussed the neurological deterioration of patients with benign tumors such as low-grade-gliomas during pregnancy [12] which can be due to hormonal changes and increased peritumoral edema caused by fluid retention and increased intravascular volume during pregnancy [7]. Such complications can, in turn, increase intracranial pressure and make early surgical interventions inevitable in pregnant patients. For example, Giannini and Bricci [55] reported a case of a 30 year old patient with cerebellopontine angle meningioma who underwent surgical resection at 25 weeks of pregnancy due to worsening neurological deficits despite the initial plan for postponed surgery and Cesarean section at 35 weeks. However, it should be emphasized that comparing a sizeable cerebellopontine angle meningioma which causes perifical edema with a newly diagnosed low-grade glioma with no mass effect or edema can be misleading. As it has been mentioned, waiting for the fetus to reach a mature age for delivery may probably not make remarkable changes to the outcome of the latter patient. Indeed, future AC studies are required to address these caveats.

It should be noted that the preferred management is to defer the surgical intervention to later stages of pregnancy accompanied by Cesarean section for stable patients. Previous studies suggested that pregnancy can proceed to the second trimester and neurosurgical procedures be performed at this stage if pathology is diagnosed in the first and early second trimester and the patient is stable [39]. However, emergent neurosurgical interventions are required if the pregnant patient is unstable [56]. The decision to proceed with operative intervention is also influenced by the stage of the pregnancy. Based on the algorithm presented by Abd-Elsayed et. al. [39] for stable symptomatic patients in the first and early second trimester, neurosurgery is postponed to the early second trimester to permit gestational advancement, with the potential use of adjuvant radiotherapy beyond the first trimester, while the operative intervention is recommended at this stage for unstable patients. Towards the end of the second trimester and early third trimester, the stable patient is observed carefully without intervention, however, if the neurological symptoms worsen, radiotherapy can be used to delay the surgery with a decision to proceed to Caesarean section followed by neurosurgical operation under general anaesthesia if the patient is unstable and there is a chance of herniation. Regardless of the gestational age, surgeries should never be delayed or denied to pregnant women according to the recommendations made by The American College of Obstetricians and Gynecologists in conjunction with the American Society of Anesthesiologists to avoid adverse consequences on the pregnant woman and fetus [57]. Neurosurgical interventions can dramatically affect the pregnant person’s physiology. Furthermore, the impact of anesthetic medications on the patient and fetus should be carefully considered. Multiple factors, including type, size, and location of the brain pathology, neurological symptoms, fetus age, and mother well-being should be considered before neurosurgical interventions during pregnancy. AC is associated with shorter hospitalization, fewer neurological deficits, improved psychological outcomes, and better prognosis and can be utilized in low-resource settings in combination with technological advances for general neurosurgical patients [21, 58–60]. AC has been used for various surgical indications, including tumor [20, 61], epilepsy [62, 63], and aneurysms [31] to enhance general neurosurgical patients' outcomes [26, 64]. Treatment strategy for AC in pregnant patients can be extrapolated from the standard of care with additional consideration.

The majority of the studies reported in this systematic review have reported the proximity to the eloquent areas as an indication to choose AC over GA, which is the main indication for AC even in the non-pregnant population. Nonetheless, avoiding the potential harm of general anesthesia to both patient and fetus, such as metabolic changes caused by prolonged propofol exposure in long operations and shorter time of the operations are listed as other indications to choose AC for pregnant patients. However, some studies have reported that the usage of propofol in shorter operations is still clinically acceptable [65]. Shorter operation time, faster postoperative recovery, and fewer complications are other factors which favor AC compared to GA in general neurosurgical patients. The positive outcomes for both the patient and fetus reported in this study suggest that AC can be considered as a safe procedure in treating pathologies in the vicinity of the eloquent areas of the brain.

Various strategies should be employed to support the physical and mental well-being of the patient to deliver a healthy and viable child. Given the presence of the fetus, patient positioning during the procedure can also be important to provide comfort for the patient as well as make the procedure feasible and reduce complications. Indeed, pregnant patient positioning during AC requires adjustments, such as placing in a semi-lateral position, depending on tumor location as well as maintaining vena cava blood flow to reduce risks for the fetus [66]. Some studies have specifically indicated that a 15° left lateral tilt should be employed for pregnant patients undergoing surgery beyond 18–20 weeks of gestation to reduce aortocaval compression and supine hypotension syndrome [33]. Specifically in AC, Pawlik et al. [46] positioned patients semi-laterally to reduce the risk of aortocaval complications [67, 68].

General neurosurgical patients diagnosed with cancer, especially brain tumor, are at risk of increased anxiety, depression, suicidal ideation and attempt [69–71]. In addition to the psychological consequences associated with diagnosis, undergoing neurosurgical interventions can cause physiological and psychological pressure on patients as well [72]. Therefore, every effort should be employed to alleviate the psychological sequelae of treatment on pregnant patients. AC has been shown to result in a lower psychological sequel for non-pregnant patients, even in low-resource settings [21, 58]. However, there have been no studies specifically evaluating the psychological well-being of pregnant patients undergoing AC, and future studies are required to address this gap in our knowledge given the increased psychological pressure that pregnant patients experience by worrying about their own as well as their fetus’s health. Furthermore, ethical considerations should be addressed regarding the well-being of the patient and the maintenance of the pregnancy or preterm termination [73, 74].

The decision to use fetal monitoring intraoperatively during AC should be based on fetal viability (generally 23 weeks and above), the feasibility of performing intraoperative monitoring, the availability of an obstetrician, the ability to easily access the maternal abdomen and safely interrupt the procedure to perform an emergency cesarean and, finally, the equipment and staff to care for the neonate [4].

Propofol is commonly used alone or in combination with a short-acting opioid for sedation during AC [75]; however, its use has been contraindicated in pregnancy due to metabolic acidosis effects [76]; Kamata et al. [44] used sevoflurane, which deteriorates intraoperative neurophysiological monitoring, instead of propofol. Adjuvant therapies post-operatively also require careful consideration of the consequences during the lactation period. For example, Kamata et al. [44] commenced adjuvant therapy when the lactation period was completed.

According to the European Society of Medical Oncology clinical practice guidelines on systemic therapy in pregnancy, chemotherapy should be avoided during the first trimester due to the high risk of congenital malformations in nearly 20% of patients [48]. Termination of pregnancy should be considered in pregnant patients with aggressive malignancies requiring chemotherapy in the first trimester [66]. Administration of most chemotherapeutic agents may be safe during subsequent trimesters; however, they may cause complications, including intrauterine growth impairment, low birth weight, premature birth, stillbirth, myocardial toxicity, and myelosuppression; therefore, their risk should be assessed for individual patients [66]. Specifically, vinblastine is safe during the first trimester, whereas antimetabolites cytarabine and 5-fluorouracil, alkylating agents cyclophosphamide and dacarbazine, anthracyclines doxorubicin and epirubicin, vinca alkaloids vinblastine, vincristine and vinorelbine, taxanes paclitaxel and docetaxel, and platinum agents cisplatin and carboplatin are safe during second and third trimesters [66, 77]. Of note, atrial septal defect, intrauterine growth restriction, preterm delivery, pre-eclampsia, and death have been reported for the pregnant patients being exposed to either vincristine or vinblastine [78]. Furthermore, six pregnant patients with brain gliomas who received either temozolomide or the combination of procarbazine, lomustine, and vincristine in the first month of the pregnancy unintentionally delivered healthy babies [79]. Temozolomide is a category D pregnancy medication which is not generally recommended, but it can be used if the benefits to the mother outweigh the risks to the fetus. However, healthy fetus delivery despite using temozolomide in the first trimester is reported [80, 81]. There are other particular considerations; for example, a three-week gap between the last chemotherapy and delivery should be made to avoid pre-term or spontaneous delivery [66, 82].

Vaginal delivery is permitted at term in a stable pregnant patient undergoing neurosurgical interventions [83], as some studies have debated that the cesarian section does not provide significant advantages over a vaginal delivery for intracranial pressure [39]. Preparations are required in case an emergency cesarian section is required. An emergency cesarian section may be required under epidural or general anesthesia if fetal distress occurs during AC [41]. Various problems, such as intraoperative seizures, emergency intubation and conversion to general anesthesia, intracranial hypertension, hemorrhage, and lack of cooperation can arise during AC, further complicating the procedure. Furthermore, hyperventilation should be avoided, which may reduce the fetus's cardiac output due to diminished venous return. Further studies are required to assess the long-term developmental consequences of exposure to AC. While insufficient information on post-operative management was provided in the articles reviewed here, the post-operative care for pregnant patients undergoing neurosurgical interventions is similar to that of non-pregnant patients [84]. However, additional intermittent fetal monitoring may be required during the post-operative phase [66].

### Limitations

Our review is subject to some limitations. There is a paucity of data in the published literature on AC during pregnancy, limiting included articles to case reports with nine patients and ten fetuses, which can affect the level of the evidence available and the strength of analyses conducted. The included articles were in English only. No study had a long-term follow-up of patients and fetuses to investigate the consequence of AC. A direct comparison of AC and general anesthesia is lacking in the literature. Furthermore, some information, such as operation time and post-operative treatment was not reported in some studies, making comparisons of the procedure with general anesthesia challenging. Despite such limitations, this article can add to the collective, yet limited, body of knowledge on neurosurgical interventions for the treatment of pregnant patients under awake conditions.

## Conclusion

AC is a safe and effective method for the treatment of lesions in pregnancy, which requires an experienced multi-disciplinary team with the support of obstetrics-gynecology. Individualized plans and decisions are required for each pregnant patient considering multiple factors, including lesion pathology, gestational age, and patient preferences. Good knowledge of the variable anesthetic agents and their effects on the fetus is very important in managing those patients. Specific guidelines should be established for the management of pregnant patients via AC.

### Supplementary Information

Below is the link to the electronic supplementary material.

Supplementary file1 (DOCX 19 KB)

## Data Availability

Not applicable.
