# Supplementary material for: Awake craniotomy during pregnancy: A systematic review of the published literature
Source: Neurosurg Rev. 2023 Nov 1;46(1):290. doi: 10.1007/s10143-023-02187-x (PMC10620271; doi:10.1007/s10143-023-02187-x)
Supplement: Supplementary file 1 — Supplementary file1 (DOCX 19 KB) [file 10143_2023_2187_MOESM1_ESM.docx]

**Supplementary Table 1.** An Overview of Search Terms Used for Each Database

| **Database** | **Search Results** | **Search Terms** |
| --- | --- | --- |
| **PubMed** | 3832 | (("awake"[All Fields] OR "awakeness"[All Fields] OR "awakes"[All Fields] OR "awaking"[All Fields]) AND ("craniotomy"[MeSH Terms] OR "craniotomy"[All Fields] OR "craniotomies"[All Fields])) OR (("awake"[All Fields] OR "awakeness"[All Fields] OR "awakes"[All Fields] OR "awaking"[All Fields]) AND ("brain"[MeSH Terms] OR "brain"[All Fields] OR "brains"[All Fields] OR "brain s"[All Fields]) AND ("surgery"[MeSH Subheading] OR "surgery"[All Fields] OR "surgical procedures, operative"[MeSH Terms] OR ("surgical"[All Fields] AND "procedures"[All Fields] AND "operative"[All Fields]) OR "operative surgical procedures"[All Fields] OR "general surgery"[MeSH Terms] OR ("general"[All Fields] AND "surgery"[All Fields]) OR "general surgery"[All Fields] OR "surgery s"[All Fields] OR "surgerys"[All Fields] OR "surgeries"[All Fields])) OR (("awake"[All Fields] OR "awakeness"[All Fields] OR "awakes"[All Fields] OR "awaking"[All Fields]) AND ("neurosurgery"[MeSH Terms] OR "neurosurgery"[All Fields] OR "neurosurgeries"[All Fields] OR "neurosurgery s"[All Fields] OR "neurosurgical procedures"[MeSH Terms] OR ("neurosurgical"[All Fields] AND "procedures"[All Fields]) OR "neurosurgical procedures"[All Fields])) OR (("awake"[All Fields] OR "awakeness"[All Fields] OR "awakes"[All Fields] OR "awaking"[All Fields]) AND ("brain mapping"[MeSH Terms] OR ("brain"[All Fields] AND "mapping"[All Fields]) OR "brain mapping"[All Fields]) AND ("awake"[All Fields] OR "awakeness"[All Fields] OR "awakes"[All Fields] OR "awaking"[All Fields]) AND ("cysts"[MeSH Terms] OR "cysts"[All Fields] OR "cyst"[All Fields] OR "neurofibroma"[MeSH Terms] OR "neurofibroma"[All Fields] OR "neurofibromas"[All Fields] OR "tumor s"[All Fields] OR "tumoral"[All Fields] OR "tumorous"[All Fields] OR "tumour"[All Fields] OR "neoplasms"[MeSH Terms] OR "neoplasms"[All Fields] OR "tumor"[All Fields] OR "tumour s"[All Fields] OR "tumoural"[All Fields] OR "tumourous"[All Fields] OR "tumours"[All Fields] OR "tumors"[All Fields]) AND ("resect"[All Fields] OR "resectability"[All Fields] OR "resectable"[All Fields] OR "resectates"[All Fields] OR "resected"[All Fields] OR "resecting"[All Fields] OR "resection"[All Fields] OR "resectional"[All Fields] OR "resectioned"[All Fields] OR "resectioning"[All Fields] OR "resections"[All Fields] OR "resective"[All Fields] OR "resects"[All Fields])) OR (("craniotomy"[MeSH Terms] OR "craniotomy"[All Fields] OR "craniotomies"[All Fields]) AND ("awake"[All Fields] OR "awakeness"[All Fields] OR "awakes"[All Fields] OR "awaking"[All Fields])) |
| **Scopus** | 4750 | ALL ( awake  AND  craniotomy  OR  awake  AND  brain  AND  surgery  OR  awake  AND  neurosurgery  OR  awake  AND  brain  AND  mapping  AND  awake  AND  tumour  AND  resection  OR  craniotomy  AND  while  AND  awake ) |
| **Web of Science** | 3600 | ALL=(awake craniotomy OR awake brain surgery OR awake neurosurgery OR awake brain mapping OR awake tumour resection OR craniotomy while awake) |

**Supplementary Table 2.** An Overview of Studies Included

| **Study** | **Article title** | **Journal** | **Country** | **Study period** | **Objective** |
| --- | --- | --- | --- | --- | --- |
| **Abd-Elsayed et al., 2013 [39]** | A case series discussing the anaesthetic management of pregnant patients with brain tumours | F1000Research | USA | May 2003 - Jun 2008 | To characterize the current perioperative management and to suggest evidence-based guidelines for the anesthetic management of pregnant females with brain tumors. |
| **Handlogten et al., 2015 [40]** | Dexmedetomidine and mannitol for awake craniotomy in a pregnant patient | Anesthesia & Analgesia | USA | NS | To describe the effects of dexmedetomidine for AC, as well as quantifying the effects of mannitol on intrauterine volume and fetal well-being. |
| **Meng et al., 2016 [41]** | Awake brain tumor resection during pregnancy: Decision making and technical nuances | Journal of Clinical Neuroscience | USA | NS | To discuss perioperative decision making and technical considerations for AC for tumor resection during pregnancy. |
| **Hedayat et al., 2017 [42]** | Awake Craniotomy for the treatment of a cortical pseudoaneurysm in a pregnant patient | Cureus | USA | NS | To discuss the management of a pseudoaneurysm in a pregnant patient treated with AC. |
| **Kamata et al., 2017 [44]** | A case of left frontal high-grade glioma diagnosed during pregnancy | JA Clinical Reports | Japan | NS | To study treatment of high-grade glioma with AC in a pregnant patient. |
| **Al Mashani, 2018 [45]** | Awake Craniotomy during pregnancy | Journal of Neurosurgical Anesthesiology | Oman | NS | To investigate AC for tumor removal in a pregnant patient. |
| **Pawlik et al., 2018 [46]** | Optimization of anaesthesia care for “total” awake craniotomy with brain tumour resection in a pregnant patient: A case report with a review of the literature | Anestezjologia | Poland | NS | To study total AC with intra-procedural speech mapping for tumor removal in a pregnant patient. |
| **Kumar et al., 2020 [47]** | Utilization of awake craniotomy for supra-tentorial tumor resection during pregnancy: A technique useful for fetal-maternal wellbeing | Pakistan Journal of Medical Science | Pakistan | NS | To describe techniques used for AC for supra-tentorial tumor resection during pregnancy. |
| **Gunasekaran et al., 2022 [43]** | Case report: Awake craniotomy during pregnancy for resection of glioblastoma | Clinical Neurology and Neurosurgery | USA | NS | To describe the management for AC for resection of glioblastoma in a pregnant patient. |

AC, awake craniotomy; NS, not specified
